# The production of fibroblast growth factor 23 is controlled by TGF-β2

**DOI:** 10.1038/s41598-017-05226-y

**Published:** 2017-07-10

**Authors:** Martina Feger, Philipp Hase, Bingbing Zhang, Frank Hirche, Philipp Glosse, Florian Lang, Michael Föller

**Affiliations:** 1 Institute of Agricultural and Nutritional Sciences, Martin-Luther University Halle-Wittenberg, D-06120 Halle (Saale), Germany; 2 Department of Physiology, Eberhard-Karls University of Tübingen, D-72076 Tübingen, Germany; 3 College of Life Science and Technology, Heilongjiang Bayi Agricultural University, Daqing, 163319 China

## Abstract

Transforming growth factor-β (TGF-β) is a cytokine produced by many cell types and implicated in cell growth, differentiation, apoptosis, and inflammation. It stimulates store-operated calcium entry (SOCE) through the calcium release-activated calcium (CRAC) channel Orai1/Stim1 in endometrial Ishikawa cells. Bone cells generate fibroblast growth factor (FGF) 23, which inhibits renal phosphate reabsorption and 1,25(OH)_2_D_3_ formation in concert with its co-receptor Klotho. Moreover, Klotho and FGF23 counteract aging and age-related clinical conditions. FGF23 production is dependent on Orai1-mediated SOCE and inflammation. Here, we explored a putative role of TGF-β2 in FGF23 synthesis. To this end, UMR106 osteoblast-like cells were cultured, *Fgf23* transcript levels determined by qRT-PCR, FGF23 protein measured by ELISA, and SOCE analyzed by fluorescence optics. UMR106 cells expressed TGF-β receptors 1 and 2. TGF-β2 enhanced SOCE and potently stimulated the production of FGF23, an effect significantly attenuated by SB431542, an inhibitor of the transforming growth factor-β (TGF-β) type I receptor activin receptor-like kinases ALK5, ALK4, and ALK7. Furthermore, the TGF-β2 effect on FGF23 production was blunted by SOCE inhibitor 2-APB. We conclude that TGF-β2 induces FGF23 production, an effect involving up-regulation of SOCE.

## Introduction

Transforming growth factor β (TGF-β) is a cytokine, which is produced by a broad range of cells^1^. It impacts on cell proliferation, growth, differentiation, migration, and apoptosis. TGF-β induces cell cycle arrest in normal cells. In cancer cells mutations of the gene encoding TGF-β may result in a loss of its anti-proliferative activity^2^. Moreover, TGF-β promotes fibrosis of many organs including the heart and kidney^1^. Its role is particularly well established in chronic kidney disease^3^. The cellular effects of TGF-β are mediated by membrane receptors including TGF-β receptors 1 and 2^4^. When binding TGF-β, the two receptors form a heterodimer and phosphorylate cellular proteins^4^.

The hormone fibroblast growth factor 23 (FGF23) is a powerful regulator of several renal functions^5–8^: It is produced by osteocytes and osteoblasts^9^. FGF23 inhibits renal phosphate reabsorption by stimulating the internalization of NaPi-IIa, the main renal Na^+^-dependent phosphate transporter^5^. In addition, it inhibits CYP27B1 or 25-hydroxyvitamin D_3_ 1-alpha-hydroxylase, the renal key enzyme for the synthesis of 1,25(OH)_2_D_3_ or calcitriol, the active form of vitamin D^5^. The consequence of these effects is a decrease of the plasma concentrations of both, phosphate and 1,25(OH)_2_D_3_. The major renal effects of FGF23 are mediated by a membrane receptor which requires the transmembrane protein Klotho as a co-receptor^5^. In addition, FGF23 has extrarenal effects, including the Klotho-independent induction of left ventricular hypertrophy^10^.

Analysis of transgenic mice has revealed a powerful influence of the two proteins on aging. Mice deficient in either FGF23 or Klotho age rapidly, have a life span of a few weeks only, and suffer from a plethora of aging-associated diseases including muscle, skin, neuronal, metabolic, and cardiovascular abnormalities^11, 12^. Notably, the aging of the mice can be almost normal if the mice are fed a low vitamin D or low phosphate diet pointing to a hitherto underestimated impact of phosphate metabolism on aging and age-associated diseases^13^.

Regulation of FGF23 formation is still incompletely understood. Recent research suggests that the plasma FGF23 concentration is a valuable and sensitive biomarker in several acute and chronic disorders^14^. In line with this the plasma FGF23 level correlates with disease activity in renal, cardiovascular, metabolic, and inflammatory diseases^14^.

Known regulators of FGF23 production include dietary phosphorus intake, parathyroid hormone (PTH)^15^, 1,25(OH)_2_D_3_^16^, the iron status^17^, and inflammation^17^. In detail, activation of the key inflammatory transcription factor NFκB up-regulates the calcium release-activated calcium (CRAC) channel Orai1/STIM1 which mediates store-operated calcium entry (SOCE)^18^. Orai1/STIM1-mediated SOCE then triggers FGF23 production. Of note, TGF-β has also been shown to up-regulate Orai1/STIM1 in endometrial tumor cells^19^. Moreover, the production of TGF-β is also enhanced in some of the aforementioned disorders associated with high serum FGF23 levels (e.g. chronic kidney disease^3^).

The present study addressed the question whether the TGF-β-isoform TGF-β2 is a regulator of FGF23 formation.

## Results

### TGF-β2 induced Fgf23 transcription in UMR106 cells

**Figure 1:**
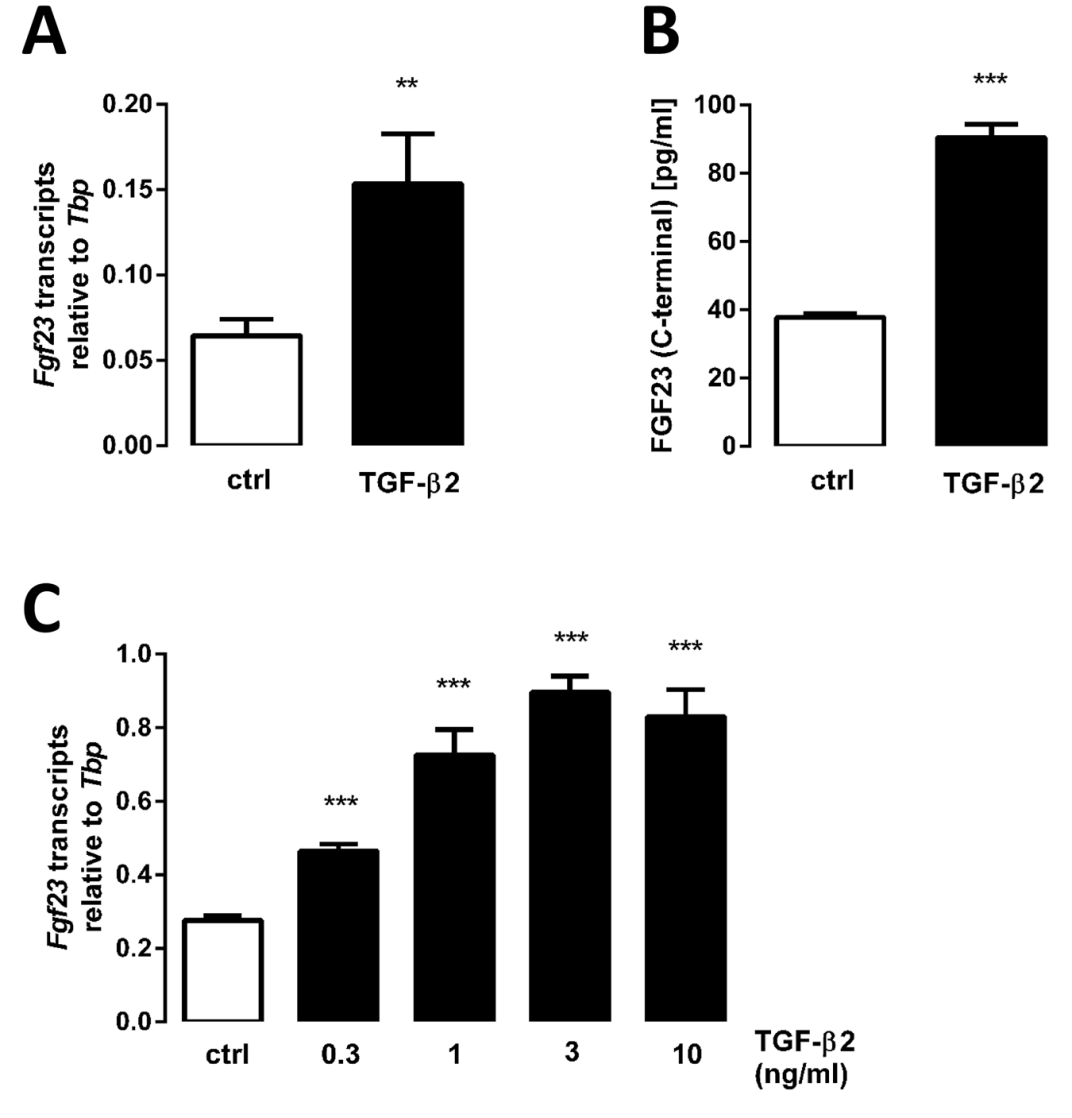
TGF-β2 stimulates *Fgf23* gene expression and FGF23 protein synthesis in UMR106 cells. Arithmetic means ± SEM (n = 7) of (**A**) *Fgf23* mRNA abundance (relative to *Tbp* mRNA) and (**B**) FGF23 (C-terminal) protein concentration (pg/ml) in the cell culture medium of UMR106 cells incubated without (ctrl, white bars) or with TGF-β2 (black bars, 10 ng/ml, 24 h). **p < 0.01, ***p < 0.001 indicate significant difference compared to UMR106 cells treated with vehicle only (paired *t*-test). (**C**) Arithmetic mean ± SEM (n = 5) of *Fgf23* mRNA levels (relative to *Tbp* mRNA) in UMR106 cells after 24-hour treatment without (ctrl, white bar) or with TGF-β2 (0.3–10 ng/ml, black bars). ***p < 0.001 indicates significant difference compared to UMR106 cells treated with vehicle only (ANOVA).

Our first series of experiments aimed to explore whether TGF-β2 is capable of influencing the production of FGF23. To this end, we treated UMR106 osteoblast-like cells with and without TGF-β2 for 24 h and determined *Fgf23* transcripts by qRT-PCR to estimate FGF23 synthesis. Figure 1A illustrates that the presence of TGF-β2 significantly up-regulated *Fgf23* transcript levels, pointing to enhanced FGF23 production. Next, we sought to investigate whether the increase in *Fgf23* transcript levels upon incubation with TGF-β2 indeed translated into enhanced FGF23 protein formation. Thus, we determined C-terminal FGF23 protein in the supernatant retrieved from cells incubated with and without TGF-β2 for 24 h using ELISA. As shown in Fig. 1B, TGF-β2 significantly stimulated FGF23 production. Figure 1C depicts the dose response effect of TGF-β2 on *Fgf23* transcripts in UMR106 cells.

### The TGF-β2 effect on Fgf23 expression was mediated by downstream TGF-β receptor signaling

**Figure 2:**
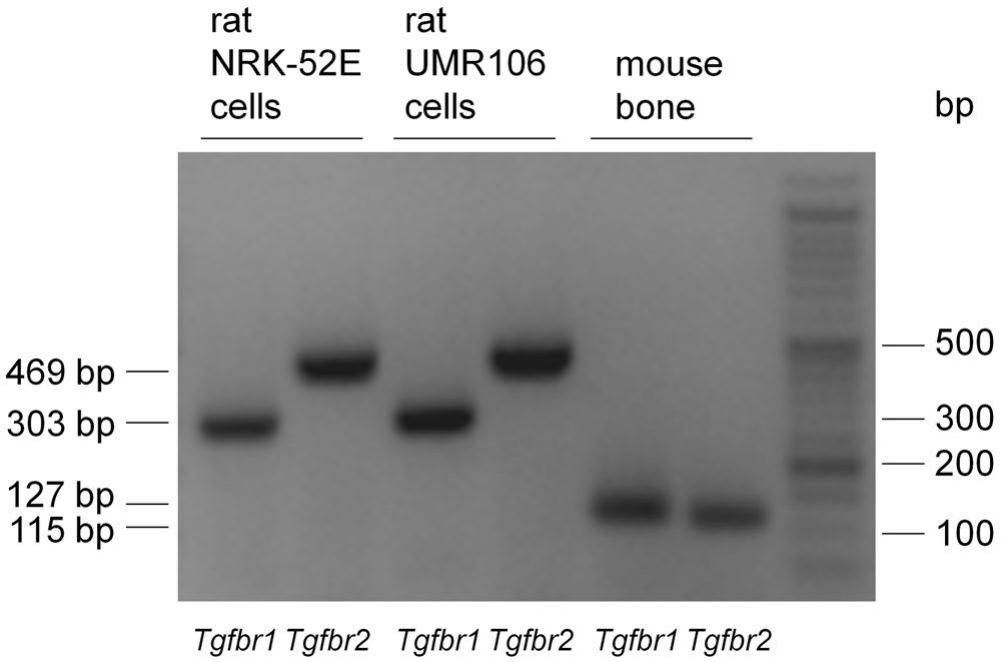
Expression of TGF-β receptor 1 and 2 in UMR106 cells and murine bone tissue. Original agarose gel photo demonstrating amplified *Tgfbr1* and *Tgfbr2*-specific cDNA in UMR106 cells and murine bone tissue. NRK-52E cells were used as a positive control.

The cellular effects of TGF-β2 are typically mediated by the joint action of TGF-β receptors 1 and 2. Employing RT-PCR, we therefore studied the expression of these receptors in UMR106 cells. It is demonstrated in Fig. 2 that UMR106 cells expressed TGF-β receptors 1 and 2. Moreover, cells retrieved from mouse bone also expressed both receptors (Fig. 2). As a control, expression of TGF-β receptors 1 and 2 in rat NRK-52E cells is provided (Fig. 2).

**Figure 3:**
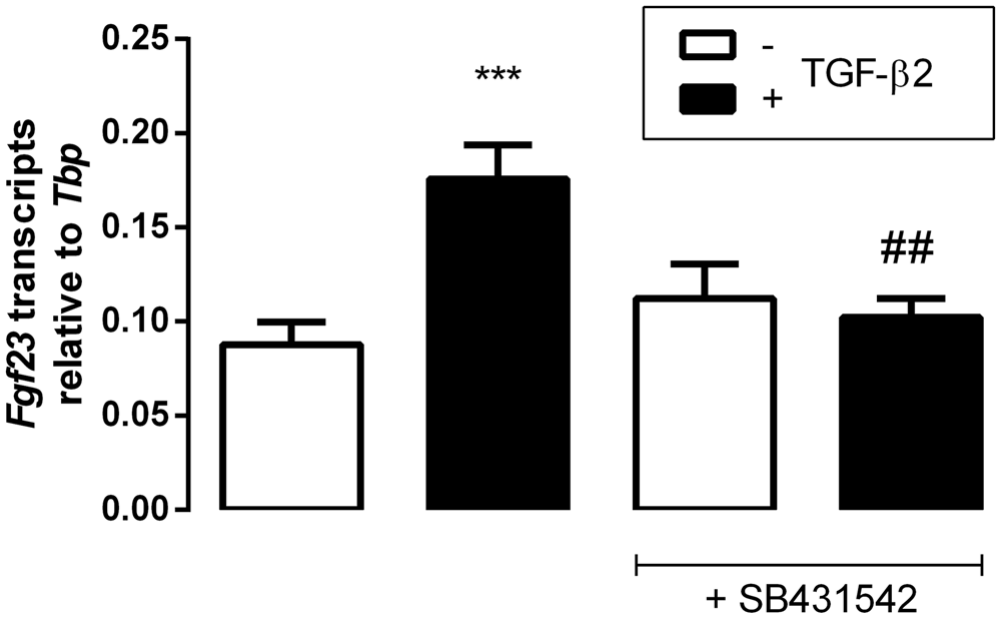
Effect of TGF-β2 on *Fgf23* transcript levels in UMR106 cells is blunted by TGF-β receptor-related kinase inhibitor SB431542. Arithmetic means ± SEM (n = 9) of *Fgf23* mRNA abundance (relative to *Tbp* mRNA) in UMR106 cells incubated without (white bars) or with (black bars) TGF-β2 (10 ng/ml) in the absence or presence of downstream TGF-β receptor-related kinase inhibitor SB431542 (10 μM) for 24 h. ***p < 0.001 indicates significant difference between cells treated with or without TGF-β2. ^##^ *p* < 0.01 indicates significant difference between cells treated with TGF-β2 alone (2^nd^ bar) and cells treated with TGF-β2 and SB431542 (4^th^ bar) (ANOVA).

As a next step, we explored whether the stimulating effect of TGF-β2 on the formation of FGF23 was dependent on downstream TGF-β receptor signaling which involves TGF-β receptor-related kinases. Hence, we determined the TGF-β2 effect on *Fgf23* transcripts in the presence and absence of SB431542, an antagonist of TGF-β receptor-related kinases. As shown in Fig. 3, the effect of TGF-β2 on *Fgf23* mRNA was significantly attenuated in the presence of SB431542. This result demonstrates that TGF-β receptor-related kinases were required for TGF-β2 to enhance FGF23 generation.

### Store-operated calcium entry (SOCE) was needed for the TGF-β2 effect on Fgf23 expression

**Figure 4:**
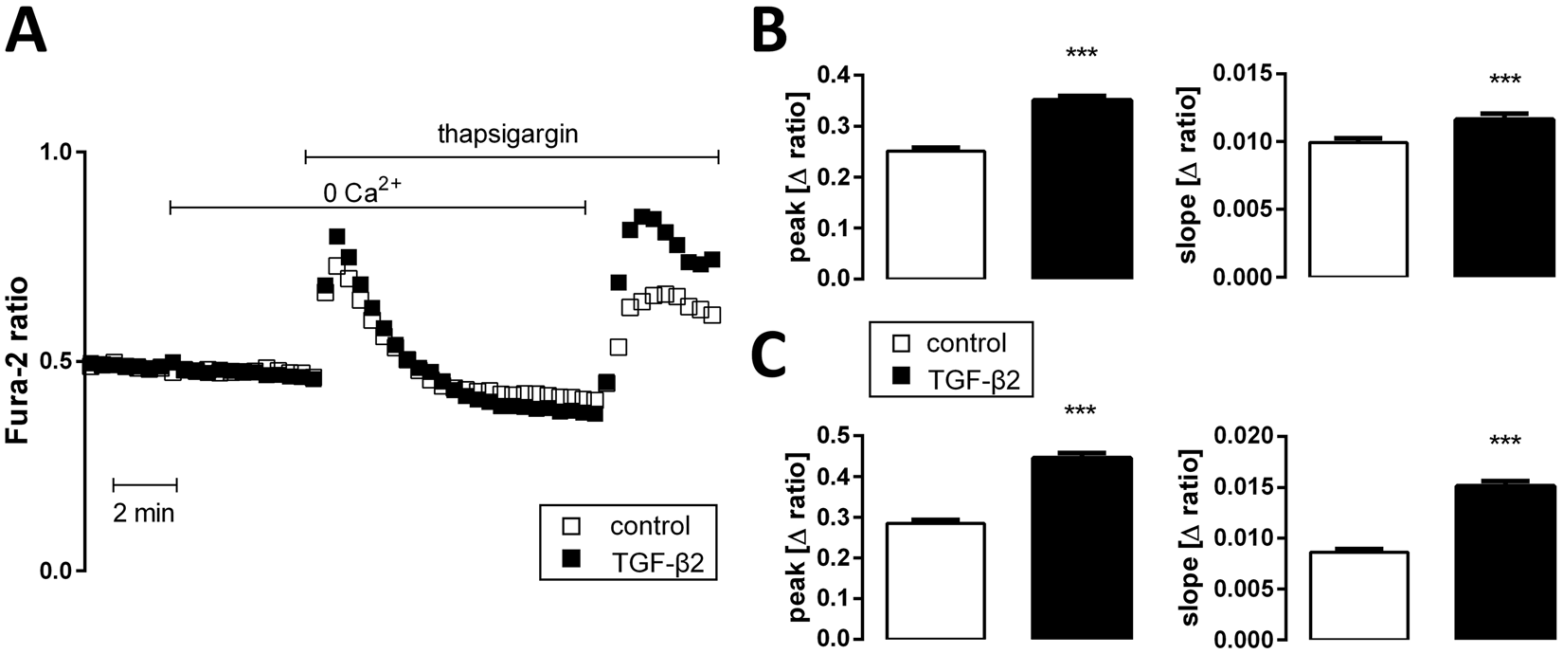
TGF-β2 induces store-operated calcium entry (SOCE) in UMR106 cells. (**A**) Representative original tracings (left panel) showing intracellular Ca^2+^ concentrations ([Ca^2+^]_i_) in Fura-2/AM-loaded UMR106 cells before and after removal of extracellular Ca^2+^, addition of the sarco-endoplasmic Ca^2+^-ATPase (SERCA) inhibitor thapsigargin (1 µM), and readdition of extracellular Ca^2+^ in the absence (open squares) and presence (closed squares) of TGF-β2 (10 ng/ml, 1 h). (**B**) Arithmetic means ± SEM (n = 124–173 cells measured on three different days) of the peak (left) and slope (right) values of [Ca^2+^]_i_ increase after addition of thapsigargin reflecting Ca^2+^ release from intracellular stores. (**C**) Arithmetic means ± SEM (n = 124–173 cells measured on three different days) of the peak (left) and slope (right) values of [Ca^2+^]_i_ increase following readdition of extracellular Ca^2+^ reflecting store-operated Ca^2+^ entry. White bars: without, black bars: with TGF-β2 (10 ng/ml, 1 h). ***p < 0.001 indicates significant difference (unpaired *t*-test).

Store-operated calcium entry through Orai1/STIM1 has been shown to induce FGF23 formation in UMR106 cells^18^. Moreover, TGF-β stimulates Orai1/STIM1 expression and thus SOCE in other cell types^19^. We therefore sought to clarify whether SOCE participates in TGF-β2-induced FGF23 release. To this end, we utilized Fura-2-dependent Ca^2+^ imaging in UMR106 cells after depletion of sarcoplasmic Ca^2+^ stores with thapsigargin to assess SOCE. As illustrated in Fig. 4, treatment with TGF-β2 enhanced the thapsigargin-induced increase of cellular Ca^2+^ and SOCE in UMR106 cells.

**Figure 5:**
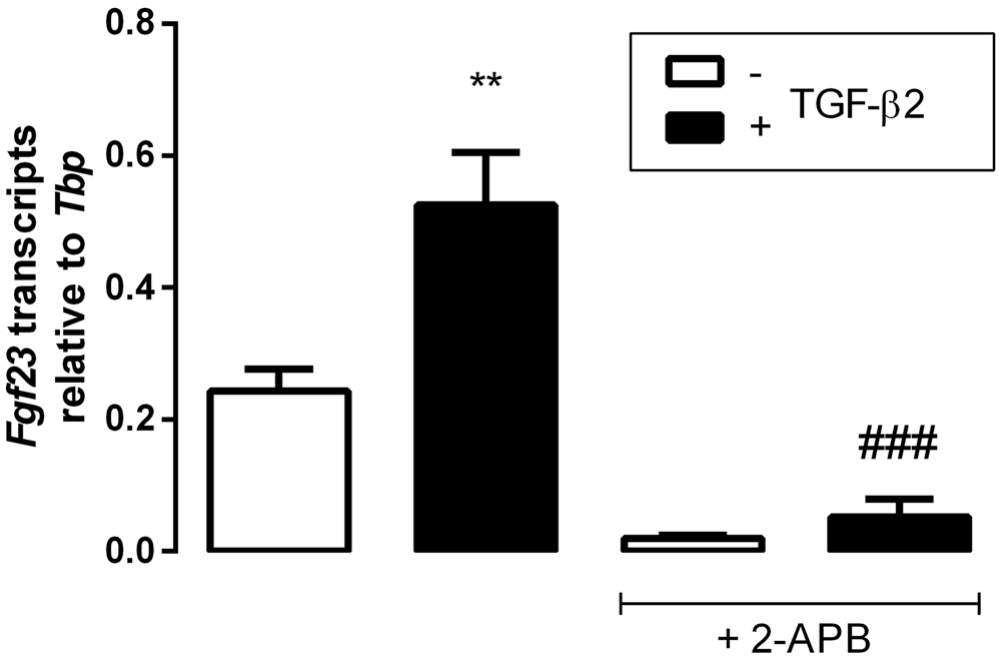
Store-operated calcium entry (SOCE) inhibitor 2-APB attenuates the TGF-β2 effect on *Fgf23* transcript levels in UMR106 cells. Arithmetic means ± SEM (n = 4) of *Fgf23* mRNA abundance (relative to *Tbp* mRNA) in UMR106 cells incubated without (white bars) or with TGF-β2 (black bars, 10 ng/ml) in the absence or presence of SOCE inhibitor 2-APB (25 μM) for 24 h. **p < 0.01 indicates significant difference from the absence of TGF-β2. ^###^ *p* < 0.001 indicates significant difference between cells treated with TGF-β2 alone (2^nd^ bar) and cells treated with TGF-β2 and 2-APB (4^th^ bar) (ANOVA).

Given the stimulating effect of TGF-β2 on SOCE in UMR106 cells, we addressed the question, whether SOCE is required for the induction of *Fgf23* transcription by TGF-β2. In order to test this, we carried out experiments with SOCE inhibitor 2-APB. According to Fig. 5, the induction of FGF23 synthesis by TGF-β2 was significantly blunted by 2-APB. This result suggests that SOCE contributes to the TGF-β2 effect on FGF23 production.

Finally we sought to explore whether the extracellular Ca^2+^ concentration influenced *Fgf23* transcription in UMR106 cells. To this end, we incubated the cells at different Ca^2+^ concentrations (adjusted by the addition of Ca^2+^ chelator EGTA or CaCl_2_, resp.) and determined *Fgf23* transcripts after 24 h. As a result, in the absence of TGF-β2 relative *Fgf23* transcripts were (n = 3 each) 0.27 ± 0.01 arbitrary units (a.u.) at 1.8 mM Ca^2+^ (control), a value not significantly different from 0 mM Ca^2+^ (0.29 ± 0.04 a.u.), 0.6 mM Ca^2+^ (0.27 ± 0.02 a.u.), 1.2 mM Ca^2+^ (0.26 ± 0.02 a.u.), 2.4 mM Ca^2+^ (0.24 ± 0.02 a.u.), and 3.0 mM Ca^2+^ (0.25 ± 0.01 a.u.).

## Discussion

According to our observations, transforming growth factor-β2 (TGF-β2) up-regulated the expression of fibroblast growth factor 23 (FGF23) in UMR106 osteoblast-like cells.

As a versatile cytokine, TGF-β is involved in a myriad of cellular effects in most tissues and organs including bone, the main site of FGF23 formation. TGF-β is required for both, the formation of bone during development as well as the maintenance and continuous remodeling throughout life^20^. Bone structures such as perichondrium, periosteum, and the epiphyseal growth plate express all TGF-β isoforms^20^. The analysis of transgenic mouse models has, however, revealed that only Tgfb2-deficient mice have an abnormal skeleton whereas Tgfb1- or Tgfb3-deficient mice do not exhibit a bone-related phenotype^20^. Moreover, it is TGF-β2 that is essential for the development of the skeleton during embryonic development^20^. Therefore, we used TGF-β2 in this study. A putative role of the other TGF-β isoforms in the regulation of FGF23 synthesis must be addressed in a future study. In later life, TGF-β is involved in bone resorption and formation and has several effects on osteoblast differentiation^20^.

Fibroblast growth factors (FGF) have already been demonstrated to be part of downstream TGF-β signaling^21^, and FGF2, FGF4, and FGF6 share the ability with TGF-ß to induce osteoblast proliferation^22^. Our study adds to these results demonstrating that bone-derived FGF23 is directly controlled by TGF-β2.

An important regulator of FGF23 formation is PTH which increases FGF23 expression^15^. Notably, TGF-β receptor 2 phosphorylates the PTH type I receptor thereby attenuating PTH signaling resulting in more trabecular and less cortical bone^23^. Thus, TGF-β is an important modulator of PTH action in bone^24^. Our present results demonstrate that the expression of FGF23, an important target of PTH, is also modulated by TGF-β.

Another major inducer of FGF23 expression is 1,25(OH)_2_D_3_, the active vitamin D metabolite. 1,25(OH)_2_D_3_ inhibits downstream TGF-β signaling^25^. Vitamin D supplementation lowers the availability of TGF-β^26^. Downstream TGF-β and 1,25(OH)_2_D_3_ signaling are characterized by intense crosstalk^27^. Our study demonstrates that TGF-β2 shares with 1,25(OH)_2_D_3_ the ability to induce FGF23 expression. It is intriguing to speculate that the regulation of FGF23 expression may also be subject to the intense 1,25(OH)_2_D_3_ – TGF-β signaling crosstalk.

Similar to what has been demonstrated in other cells types^19^, TGF-β2 induced SOCE, but also the thapsigargin-induced elevation in cytosolic Ca^2+^ in UMR106 cells. In addition, our study shows that TGF-β2-induced gene expression of *Fgf23* was sensitive to 2-APB, an inhibitor of SOCE. These results suggest that the full effect of TGF-β2 required SOCE. In line with this, UMR106 cells have been demonstrated to express Orai1/STIM^18^, and *Fgf23* gene expression is enhanced upon Orai1-mediated calcium entry^18^. Moreover, TGF-β up-regulates Orai1^19^ and induces cellular calcium influx^28^.

Our results suggest that in the absence of TGF-β2 the extracellular Ca^2+^ concentration does not *per se* influence *Fgf23* transcription in UMR106 cells. In contrast, Orai1-mediated Ca^2+^ influx from the extracellular space is required for the full TGF-β2 effect on FGF23.

Notably, the stimulating effect of advanced glycation end products (AGEs)^29^ and aldosterone^30^ on *Fgf23* transcription was also significantly blunted by SOCE inhibitor 2-APB, and NFκB has similarly been shown to induce *Fgf23* gene expression by enhancing SOCE^18^. Therefore, it appears to be possible that other regulators of SOCE similarly influence *Fgf23* transcription.

An elevated FGF23 plasma level is observed in many acute and chronic disorders^14^. Among them, the role of FGF23 has most extensively been studied in chronic kidney disease (CKD) where the FGF23 plasma concentration rises even before the plasma phosphate concentration increases^31^. Notably, CKD and CKD-associated renal fibrosis are associated with enhanced TGF-β formation^32^. Given our present results, it appears likely that enhanced TGF-β production in CKD and other disorders contributes to the high FGF23 plasma concentration common in those diseases. According to a recent study, FGF23 also influences TGF-β expression^33^. Together with our findings, this paper suggests that FGF23 and TGF-β signaling are closely intertwined.

Taken together, TGF-β2 enhanced gene expression and protein synthesis of FGF23 in osteoblast-like cells. The effect was paralleled by enhanced SOCE which was required for the full effect on *Fgf23* gene expression. TGF-β2-induced FGF23 formation presumably contributes to high plasma FGF23 levels in acute and chronic diseases.

## Methods

### Cell culture

Cell culture experiments were conducted as previously described^34^. Briefly, UMR106 rat osteoblast-like cells and NRK-52E rat tubular epithelial kidney cells were cultured in DMEM high glucose medium (Gibco, Life Technologies, Darmstadt, Germany) containing 1.8 mM Ca^2+^ supplemented with 10% FBS (Gibco, Life Technologies) (or 5% BCS (Sigma, Schnelldorf, Germany) for NRK-52E cells, resp.) and 100 U/mL penicillin/100 μg/ml streptomycin (Gibco, Life Technologies) under standard culture conditions. UMR106 cells do not have appreciable amounts of *Fgf23* mRNA *per se*. *Fgf23* expression was therefore induced by pre-treatment with 100 nM 1,25(OH)_2_D_3_ (Tocris, Bristol, UK)^35^. After 24 h, cells were additionally treated with TGF-β2 with or without 25 μM 2-APB or 10 µM SB431542 (all from Sigma) for another 24 h or treated with vehicle only. For the calcium measurements, cells were treated with or without 10 ng/ml TGF-β2 for 1 h.

Calcium concentration in the medium of pretreated UMR106 cells was modified by adding EGTA (0.6–1.8 mM) or CaCl_2_ (0.6 and 1.2 mM) for another 24 h.

### ELISA

To assess FGF23 release into the cell culture medium, UMR106 cells were treated for 24 h without or with TGF-β2 (10 ng/ml). Cell culture media were collected for subsequent FGF23 (C-terminal) measurements using an ELISA kit (Immutopics, San Clemente, CA, USA) according to the manufacturer’s protocol.

### Quantitative real-time PCR

Total RNA was extracted from UMR106 cells in Tri-Fast (Peqlab, Erlangen, Germany). Messenger RNA was transcribed with GoScript™ Reverse Transcription System (Promega, Mannheim, Germany) using 1.2 μg of total RNA and random primers. For qRT-PCR analysis, the final volume of the RT-PCR reaction mixture was 20 µl and contained: 2 µl cDNA, 1 µM of each primer, 10 µl GoTaq^®^ qPCR Master Mix (Promega), and sterile water up to 20 µl. PCR conditions were 95 °C for 3 min, followed by 40 cycles of 95 °C for 10 s, 58 °C for 30 s and 72 °C for 45 s. Quantitative RT-PCR was performed on a Rotor-Gene Q (QIAGEN, Hilden, Germany).

The following primers were used:

*Rat Tbp* (TATA box-binding protein)

forward (5′-3′): ACTCCTGCCACACCAGCC

reverse (5′-3′): GGTCAAGTTTACAGCCAAGATTCA

*Rat Fgf23*

forward (5′-3′): TGGCCATGTAGACGGAACAC

reverse (5′-3′): GGCCCCTATTATCACTACGGAG

Calculated mRNA expression levels were normalized to the expression levels of *Tbp* of the same cDNA sample as internal reference. Relative quantification of gene expression was performed using the ΔCt method.

### Qualitative expression analysis

Total RNA was extracted from UMR106 cells treated for 48 h with 100 nM 1,25(OH)_2_D_3_ only or from NRK-52E cells. For analysis of TGF-β receptor type 1 and 2 expression in murine bone tissue, femur and tibia were scrubbed removing residual soft tissue. Both epiphyses from femur and tibia were removed and the bone marrow was flushed out of the bone with 0.9% NaCl. Bone tissue was homogenized in liquid nitrogen using a mortar and pestle. Total RNA from bone was extracted with Tri-Fast (Peqlab). Next, the RNeasy Mini Kit (QIAGEN) was employed according to the manufacturer’s protocol.

PCR was carried out using 2 μl cDNA, 1 μM of each primer, 10 μl GoTaq^®^ Green Master Mix (Promega) and sterile water up to 20 μl. The PCR conditions were: 95 °C for 3 min, followed by 35 cycles of 95 °C for 30 s, annealing at primer-specific temperature (56–60 °C) for 30 s, and 72 °C for 45 s (PCR Thermocycler BiometraTone, Analytik Jena, Jena, Germany). PCR products were analyzed by electrophoresis on a 1.5% agarose gel and visualized by Midori Green (Biozym, Hessisch Oldendorf, Germany) staining.

The following primers were used:

*Rat Tgfbr1* (TGF-β receptor 1)

forward (5′-3′): CTGCCTGCTTCTCATCGTGT

reverse (5′-3′): TGCTTTTCTGTAGTTGGGAGTTCT

*Rat Tgfbr2* (TGF-β receptor 2)

forward (5′-3′): CCCAAGTCGGTTAACAGCGAT

reverse (5′-3′): TGGCAATGACAGCTATGGCA

*Mouse Tgfbr1*

forward (5′-3′): CCTGAAGTTCTAGATGATTCC

reverse (5′-3′): CTTCATGGATTCCACCAATAG

*Mouse Tgfbr2*

forward (5′-3′): CCAGGATGAATCTGGAAAAC

reverse (5′-3′): TAATCCTTCACTTCTCCCAC

### Calcium measurements

The measurements were performed as described previously^18^. Fura-2 fluorescence was utilized to estimate the cytosolic Ca^2+^ concentration ([Ca^2+^]_i_). Cells were loaded with Fura-2/AM (1 μM, Biomol, Hamburg, Germany) for 15 min at 37 °C. Fluorescence of the cells was excited alternatively at 340 nm and 380 nm (emission 510/87 nm, Fura-2 filter set) through an objective (CFI S-Fluor 40 x/1.30 oil) in an inverted microscope (Eclipse TS 100, all of Nikon, Düsseldorf, Germany) by a Lumen 220 Pro lamp (Prior Scientific Instruments, Cambridge, UK). Emitted fluorescence intensity was recorded by an Orca Flash 4.0 camera (Hamamatsu Photonics, Herrsching, Germany). System control and data acquisition were accomplished by µManager software. Cytosolic Ca^2+^ activity was estimated from the 340 nm/380 nm ratio. SOCE was determined after extracellular Ca^2+^ removal and subsequent Ca^2+^ readdition in the presence of thapsigargin (1 µM, Sigma). For quantification of Ca^2+^ entry, the slope (delta ratio/s) and peak (delta ratio) following readdition of Ca^2+^ ﻿were calculated. Experiments were performed in Ringer solution containing (in mM): 125 NaCl, 5 KCl, 1.2 MgSO_4_, 2 CaCl_2_, 2 Na_2_HPO_4_, 32 HEPES, 5 glucose, pH 7.4. To reach nominally Ca^2+^-free conditions, Ca^2+^-free Ringer solution was used (in mM: 125 NaCl, 5 KCl, 1.2 MgSO_4_, 2 Na_2_HPO_4_, 32 HEPES, 0.5 EGTA, 5 glucose, pH 7.4).

### Statistics

Data are provided as means ± SEM, *n* represents the number of independent experiments. All data were tested for significance using paired or unpaired Student’s *t*-test or one-way ANOVA. Only results with *p* < 0.05 were considered statistically significant.
